# Sex Differences in Brain Thyroid Hormone Levels during Early Post-Hatching Development in Zebra Finch (*Taeniopygia guttata*)

**DOI:** 10.1371/journal.pone.0169643

**Published:** 2017-01-06

**Authors:** Shinji Yamaguchi, Shin Hayase, Naoya Aoki, Akihiko Takehara, Jun Ishigohoka, Toshiya Matsushima, Kazuhiro Wada, Koichi J. Homma

**Affiliations:** 1 Department of Life and Health Sciences, Faculty of Pharmaceutical Sciences, Teikyo University, Kaga, Itabashi-ku, Tokyo, Japan; 2 Department of Biology, Faculty of Science, Hokkaido University, Hokkaido, Japan; Claremont Colleges, UNITED STATES

## Abstract

Thyroid hormones are closely linked to the hatching process in precocial birds. Previously, we showed that thyroid hormones in brain had a strong impact on filial imprinting, an early learning behavior in newly hatched chicks; brain 3,5,3′-triiodothyronine (T_3_) peaks around hatching and imprinting training induces additional T_3_ release, thus, extending the sensitive period for imprinting and enabling subsequent other learning. On the other hand, blood thyroid hormone levels have been reported to increase gradually after hatching in altricial species, but it remains unknown how the brain thyroid hormone levels change during post-hatching development of altricial birds. Here, we determined the changes in serum and brain thyroid hormone levels of a passerine songbird species, the zebra finch using radioimmunoassay. In the serum, we found a gradual increase in thyroid hormone levels during post-hatching development, as well as differences between male and female finches. In the brain, there was clear surge in the hormone levels during development in males and females coinciding with the time of fledging, but the onset of the surge of thyroxine (T_4_) in males preceded that of females, whereas the onset of the surge of T_3_ in males succeeded that of females. These findings provide a basis for understanding the functions of thyroid hormones during early development and learning in altricial birds.

## Introduction

Thyroid hormones are critical to embryonic development, as they control the growth and differentiation of nervous, muscular, and skeletal tissues in avian species [1, 2]. Among the several known functions of thyroid hormones, their role in the maturation of the late embryonic brain is well studied [3, 4]. Disturbances in the embryonic function of thyroid hormones due to by diseases such as perinatal hypothyroidism and thyroid hormone resistance syndrome are known to result in severe central nervous system (CNS) dysfunctions in humans and rodents [5, 6]. Apart from their involvement in the late embryonic brain development, there is little knowledge about the post-embryonic function of thyroid hormones in the brain, except for the maintenance of homeostasis. The effect of thyroid hormone on the cognitive function in rats has been previously studied; it enhances the animal’s ability to learn a spatial memory task [7]. Thyroid hormone also acts locally to increase neurogenesis and neuronal differentiation in the tadpole visual system [8].

We recently found that thyroid hormones have a crucial impact on filial imprinting, a learning behavior observed just after hatching in chickens (*Gallus gallus domesticus*) [9]. Filial imprinting is a learning process that newly hatched precocial chicks and ducklings undergo when they are exposed to the first moving object after hatching, on which they imprint and follow [10]. Usually, this moving object is their mother or sibling. In natural conditions, we previously found that the serum concentration of T_3_ and T_4_ in chicken peaked around hatching under natural conditions. In the brain, T_3_ gradually accumulated from six days before hatching, peaked around hatching, and then declined to the baseline level five days after hatching, whereas T_4_ became almost undetectable, probably because of its high turnover rate. Importantly, imprinting training triggers a further acute inflow of T_3_ in the brain, beyond its threshold level, which results from conversion of circulating T_4_ in the endothelial cells [9]. Thus, T_3_ in the brain determines the start of the sensitive period of imprinting, and exogenous T_3_ can reopen a previously closed sensitive period, thereby enabling imprinting and subsequent learning [9]. T_4_ is known to be the main hormone secreted by the thyroid gland in vertebrates, and the amount of blood thyroid hormone depends on the production rate of the thyroid gland. T_4_ is transported into the brain via transporters and converted to T_3_ by iodothyronine deiodinase (Dio2). Thus, control of the deiodinase activity is critical for maintaining the T_3_ level in the brain [11].

Several studies on altricial birds have suggested the absence of a distinct perihatch peak for the plasma thyroid hormone [12–16]. Typically, the concentration of circulating thyroid hormone increases gradually to reach levels typically recorded in adult in approximately 3–4 weeks post-hatching [16]. In contrast to precocial birds, altricial birds hatch with their eyes and ears closed and show less locomotor activity [1]. For example, passerine songbird zebra finch (*Taeniopygia guttata*) chicks open their eyes by 10 days post-hatching (dph); leave their nest around 20–25 dph; and then after fledging, they start listening and memorizing songs from their father [17]. In the case of precocial chicks, the levels of thyroid hormones in the brain reflect the corresponding levels in the serum [9]. Therefore, we assumed that brain thyroid hormone levels in zebra finch reflect the corresponding serum levels, which increase gradually and reach adult levels in a few weeks post-hatching. Here, we evaluated the post-hatching developmental changes in thyroid hormone levels of zebra finch in the brain and serum_._ These data are expected to give us a perspective on the relationship between thyroid hormones and early development in altricial species.

## Materials and Methods

### Animals

The experiments were conducted according to the guidelines of the committees on animal experiments of Teikyo University (approval number: 13–029) and Hokkaido University (approval number: 13–0068). These guidelines are based on the national regulations for animal welfare in Japan (Law for the Humane Treatment and Management of Animals; after partial amendment number 68, 2005). We used male and female zebra finches (age range: 1–40 dph) maintained in our breeding colony. The number of animals used in each experiment is shown in the Table 1 and S1 Table. The photoperiod was constantly maintained at a 13/11-hr light/dark cycle at 25°C. Avian diet consisted of dry seeds, and water was provided *ad libitum*. Birds were raised in breeding cages with their parents and siblings (2–6 birds per cage). To distinguish male chicks from females, genomic DNA was extracted from the fingers of individual chicks and used for genomic PCR analysis [18] with the following primers: sense, TTGCCAAGGATGAGAAACTG; antisense, TCTTCTCCTCCTACTGTGTT. Animals were killed by decapitation, and samples were collected 3 hr after the onset of the light period. Telencephalon, not including the thalamus, midbrain, spinal cord, and cerebellum, was rapidly removed using optical tweezers, frozen on dry ice, and stored at −80°C. Serum was separated by centrifuging the blood for 15 min at room temperature and stored at −80°C.

**Table 1:** The number of animals used in each experiment.

		1,2 dph	5 dph	10 dph	15 dph	20 dph	25 dph	30 dph	35 dph	-
Fig 1C	Male (M)	3	5	4	5	4	5	5	3	-
	Female (F)	0	4	8	6	5	5	7	4	-
	total	3	9	12	11	9	10	12	7	-
Fig 2A		1,2 dph	5 dph	10 dph	15 dph	20 dph	25 dph	30 dph	35 dph	40 dph
	(T3+T4) M	3	5	4	5	4	5	5	3	0
	(T3+T4) F	0	4	8	6	5	5	7	4	6
Fig 2B		1,2 dph	5 dph	10 dph	15 dph	20 dph	25 dph	30 dph	35 dph	40 dph
	T4 M	3	5	5	6	5	5	5	4	0
	T4 F	0	4	8	6	5	6	7	5	6
Fig 2C		1,2 dph	5 dph	10 dph	15 dph	20 dph	25 dph	30 dph	35 dph	40 dph
	T3 M	7	5	4	5	5	6	5	4	0
	T3 F	0	6	8	6	5	5	7	4	6
Fig 3A		1,2 dph	5 dph	10 dph	15 dph	20 dph	25 dph	30 dph	35 dph	40 dph
	(T3+T4) M	5	5	5	5	4	5	5	4	2
	(T3+T4) F	5	6	7	8	5	4	5	2	5
Fig 3B		1,2 dph	5 dph	10 dph	15 dph	20 dph	25 dph	30 dph	35 dph	40 dph
	T4 M	5	5	5	5	5	5	5	4	2
	T4 F	5	6	7	8	7	4	5	2	7
Fig 3C		1,2 dph	5 dph	10 dph	15 dph	20 dph	25 dph	30 dph	35 dph	40 dph
	T3 M	5	5	5	6	4	5	5	5	2
	T3 F	5	6	7	8	5	5	6	6	5

### Methanol/chloroform extraction of thyroid hormones

Thyroid hormones from zebra finch brains were extracted using methanol and chloroform as described in detail elsewhere [19]. To remove the lipid components, brains were homogenized in ice-cold methanol (4 ml for each brain sample). After centrifugation (TOMY SEIKO, Tokyo, Japan, RL-603) at 2,000 rpm (750 × *g*) for 10 min, the supernatant was stored and the precipitate was extracted again with methanol. To the combined supernatant, chloroform (14 ml) and 0.05% CaCl_2_ (4 ml) were added and mixed vigorously. After centrifugation at 3,000 rpm (1,670 × *g*) for 10 min, the upper polar layer was collected and vacuum-dried overnight at 37°C. The residue was dissolved in 1.0 ml of 0.11 M barbital buffer (pH 8.6), containing 0.1% gelatin. The extraction efficiency was generally 45–55%. About 2,000 cpm of ^125^I-labeled T_3_ (PerkinElmer Japan, Kanagawa, Japan) was added to each homogenate just before the first centrifugation as an internal standard to assess the individual recoveries. Brain T_3_ and T_4_ levels were determined by radioimmunoassay (RIA).

### Radioimmunoassay

**Fig 1:**
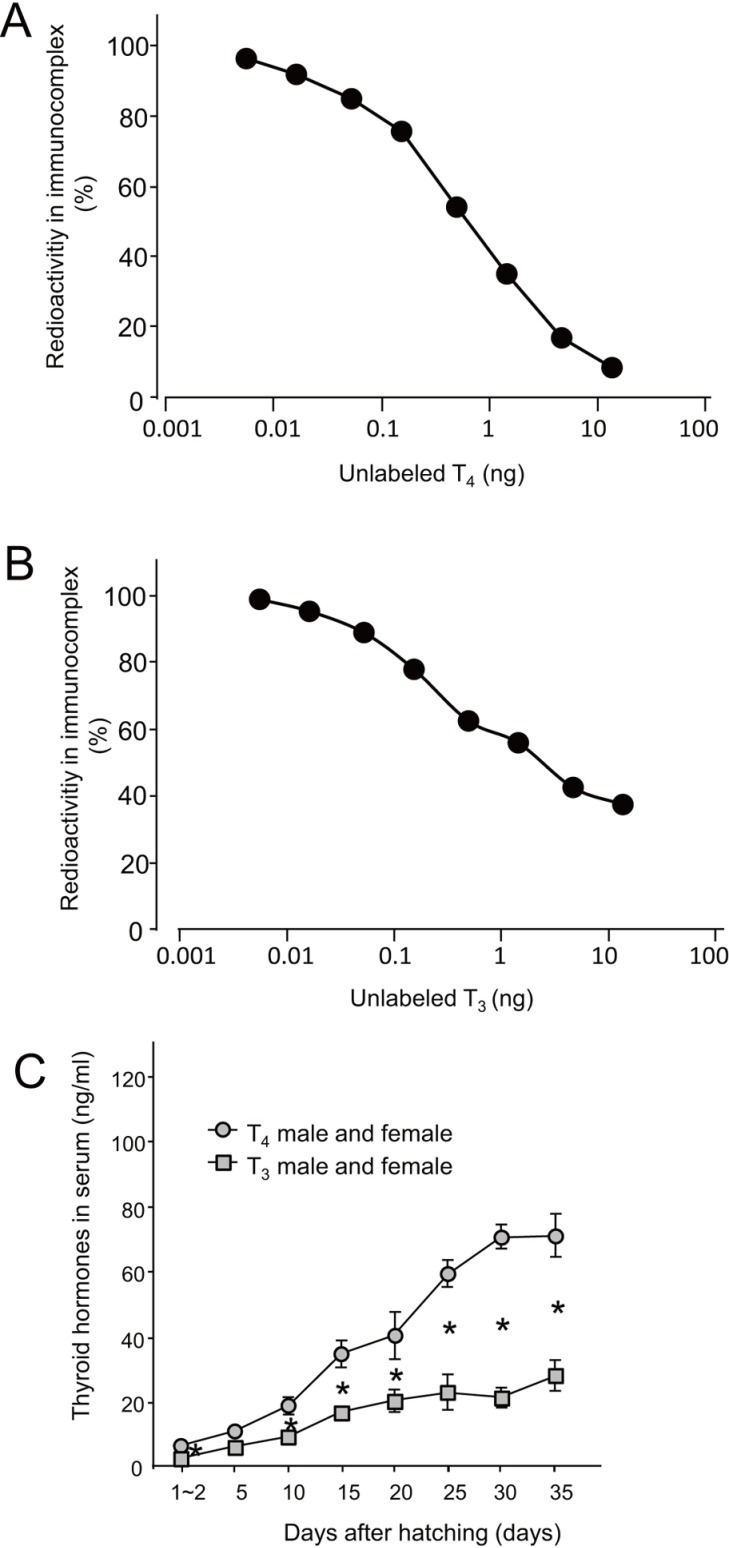
Standard curves for radioimmunoassay (RIA) and serum thyroid hormone levels in zebra finches. Typical standard curves for (A) T_4_ and (B) T_3_ are shown. Each point represents the average of duplicate measurements. Standard deviations were small and fell within the symbols. (C) Comparison of T_3_ and T_4_ in serum for males and females averaged through time post-hatching (gray ○, T_4_; gray □, T_3_)_._ A two-way repeated measures ANOVA was done following Bartlett’s test for homogeneity of variance. The significance level was set at *P* < 0.05. Mean ± SEM are reported in the graphs.

**Fig 2:**
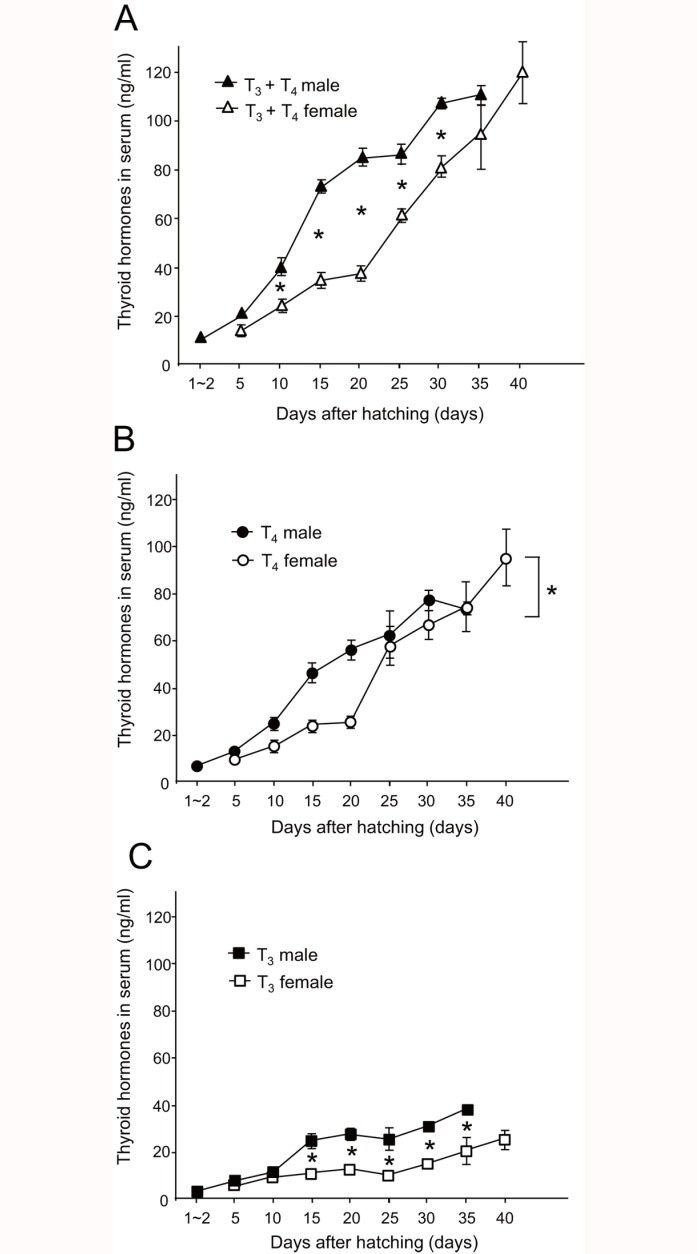
Serum thyroid hormone levels in male and female zebra finches. Comparisons between male and female zebra finches for (A) total concentrations of T_3_ and T_4_ (▲, T_3_ + T_4_ male;△, T_3_ + T_4_ female), (B) T_4_ (●, male T_4_; ○, female T_4._), and (C) T_3_ (■, male T_3_; □, female T_3_) levels through days post-hatching. A two-way factorial ANOVA was done following Bartlett’s test for homogeneity of variance. The significance level was set at *P* < 0.05. Mean ± SEM are reported in the graphs.

**Fig 3:**
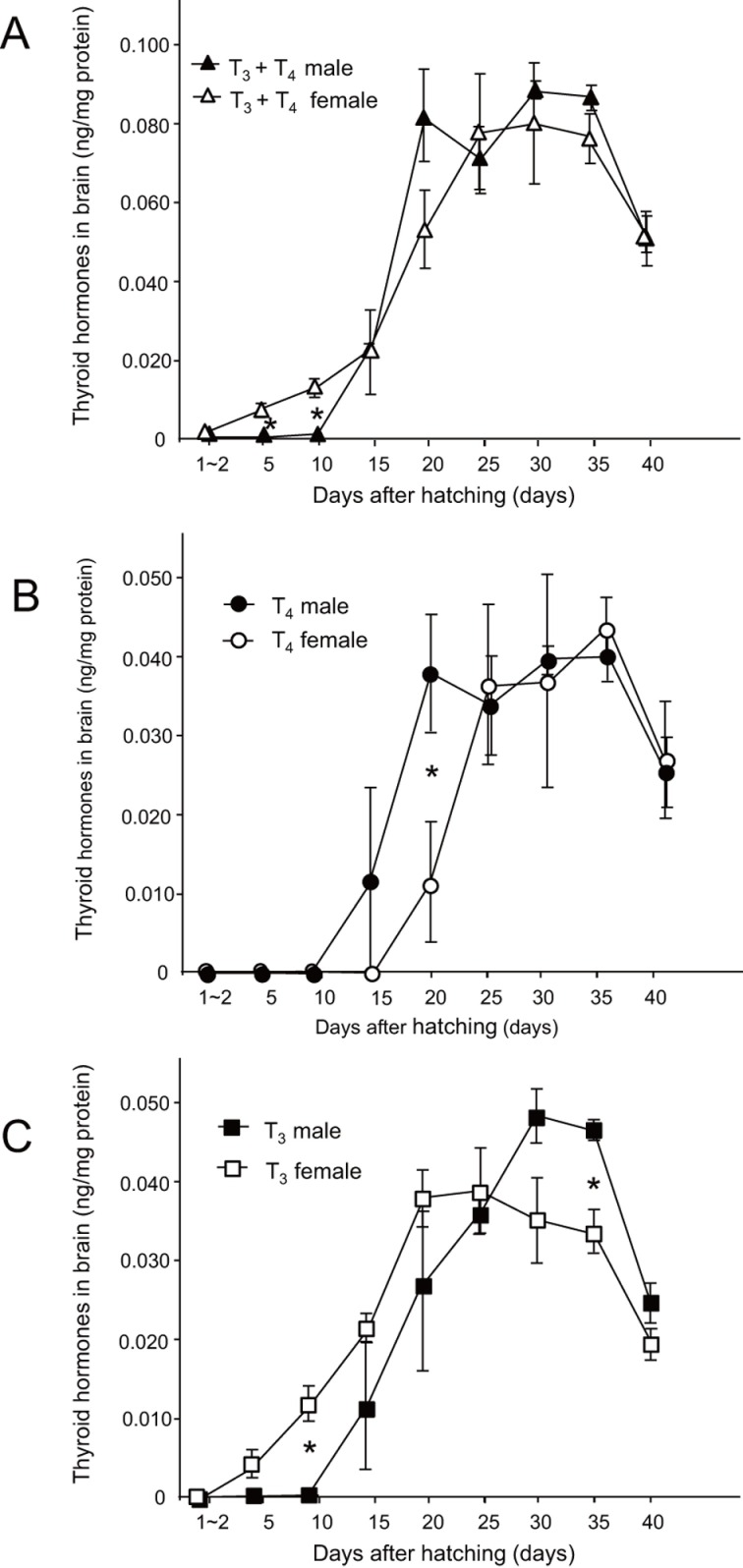
Brain thyroid hormone levels in male and female zebra finches. Comparisons of male and female brain thyroid hormones through time after hatching for (A) total concentrations of T_3_ and T_4_ (▲, T_3_ + T_4_ male; △, T_3_ + T_4_ female), (B) T_4_ (●, male T_4_; ○, female T_4._), and (C) T_3_ (■, male T_3_; □, female T_3_). Kruskal-Wallis tests were used to analyze the concentration of thyroid hormones in brains. Mann-Whitney’s U-test with Bonferroni correction was used for *post-hoc* multiple comparisons. The significance level was set at *P* < 0.05. Mean ± SEM are reported in the graphs.

Concentrations of thyroid hormones in the brain and serum of zebra finches were determined as described elsewhere [9, 20, 21]. Standard T_3_ and T_4_ were obtained commercially (Sigma-Aldrich, MO, USA). The anti-T_3_ antibody (Sigma-Aldrich, catalog # T2777) or anti-T_4_antibody (Sigma-Aldrich, catalog # T2652) was diluted in advance to a concentration that would result in the binding of 30–40% labeled T_3_ or T_4_ (PerkinElmer Japan) without the addition of unlabeled T_3_ or T_4_. Radiolabeled thyroid hormones and specific anti-serum were incubated with increasing amounts of unlabeled thyroid hormones. Each antibody was added and incubated for 2 hr on ice, in a total volume of 250 μl (T_3_, final dilutions of 1:62,500; T_4_, final dilutions of 1:16,000) and then precipitated by adding cold polyethylene glycol 6000 (Sigma-Aldrich). After incubation for 30 min on ice, the samples were centrifuged at 12,000 rpm for 30 min at 4°C. The supernatant was aspirated and the precipitates were counted using a gamma counter (AccuFLEX ARC-7000, ALOKA, Tokyo, Japan). The relative amount of radioactivity in the immunocomplex was plotted against the amount of competitor. The standard curves for T_3_ and T_4_ were plotted using the average of duplicate measurements. The amount of T_3_ or T_4_ was determined from the standard curves. The radioactivity increased linearly with the increase in the amount of unlabeled T_3_ or T_4_. For each dph, we used 4–8 male and female brains and serum samples through the RIA experiments, except for the 40-dph male brain (n = 2) and the 35-dph female brain (n = 2). Details about the numbers of animals we used are shown in Table 1 and S1 Table. Concentrations of thyroid hormones in the brain and serum samples were determined using single measurements and mean ± SEM (Fig 1C, Fig 2 and Fig 3). The relative standard error of the mean (SEM/mean) was maximum at 26.7% (serum) and 38.2% (brain), except for 15 dph in both the male and female brains. Raw data for RIA are shown in the Supporting Information files (S1 Table).

### Immunofluorescence

Brain specimens, including the thalamus, midbrain, spinal cord, and cerebellum, were rapidly collected, frozen in a plastic mold with Tissue-Tek OCT compound (Sakura Fine Technical, Tokyo, Japan), placed on dry ice, and stored at −80°C. Serial sagittal sections, 12 μm thick, were cut throughout the brain and immunofluorescence was performed as described previously [22]. Rabbit anti-thyroid hormone receptor alpha antibody (GeneTex, Irvine, CA, USA, catalog # GTX25621, 1:100) and mouse anti-NeuN monoclonal antibody (Chemicon, Darmstadt, Germany, catalog # MAB377) were used as the primary antibodies. Anti-rabbit Alexa 488-conjugated antibody (Invitrogen, CA, USA, catalog # A11008, 1:200) and anti-mouse Alexa 568-conjugated antibody (Invitrogen, catalog # A11003, 1:200) were used as the secondary antibodies. Fluorescence images of brain sections were obtained using a TCS-SP5 confocal fluorescence microscope (Leica Microsystems, Tokyo, Japan) and NanoZoomer (Hamamatsu Photonics, Shizuoka, Japan).

### Quantitative RT-PCR

Total RNA was isolated from the sagittal sections using TRIzol reagent (Invitrogen) covering the entire cerebrum regions. Total RNA (1 μg) was treated with RNase-free DNaseI (Invitrogen) and subjected to RT-PCR following the technical procedure in our previous paper [23]. The primers used were as follows: Dio2, 5′-CACACATGCACTTACACAGTTGGT-3′ (sense) and 5′-TCACTGGGACATCTGACAACTACTC-3′ (antisense); GAPDH, 5′-GCAGGACTCTCCTTTGTTGGA-3′ (sense) and 5′-GGGAGAAGTTGGAGGAGTGG -3′ (antisense). Raw data for Quantitative RT-PCR are shown in the Supporting Information files (S2 Table).

### Statistical analysis

Parametric tests (two-way repeated measures ANOVA for Fig 1C and two-way factorial ANOVA for Fig 2A, 2B and 2C) were conducted following Bartlett’s test for homogeneity of variance to analyze the concentration of thyroid hormones in serum (Fig 1C, 2A, 2B and 2C). The concentrations did not match the Gaussian distribution and, thus, the variances were not expected to be homogenous. Therefore, the concentrations determined by RIAs were transformed by taking their 2.8 root (Fig 1C), 1.4 root (Fig 2A), 3.0 root (Fig 2B) or 1.2 root (Fig 2C). The converted data could be reliably examined by parametric methods. Moreover, non-parametric tests (Kruskal-Wallis test) were used to analyze the concentration of thyroid hormones in the brain (Fig 3A, 3B and 3C) because male brain samples of 1–10 dph contained undetectable levels of thyroid hormone, and thus, the variances showed significant differences. If necessary, Mann-Whitney’s *U*-test with Bonferroni correction was used for *post-hoc* multiple comparisons. The significance level was set at *P* < 0.05. Mean ± SEM are reported in the graphs. For statistical analysis of quantitative RT-PCR, the Kruskal-Wallis test and subsequent multiple comparisons were performed. The significance level was set at *P* < 0.05. Mean was shown in the graphs as a bar.

## Results

To estimate the thyroid hormone levels in the brain and serum during post-hatching development of zebra finch, we established a RIA system. The assay was based on determining the amount of thyroid hormones that inhibited the formation of immunocomplexes [9, 20, 21]. The standard curves obtained with serial dilutions of thyroid hormones are shown in Fig 1A and 1B. We were thus able to detect thyroid hormones in each sample up to the nanogram level. We then examined the changes in serum thyroid hormone levels during post-hatching development of zebra finches. Serum T_3_ and T_4_ increased gradually with the number of days post-hatching, and T_4_ levels were higher than T_3_ levels during the course of post-hatching development (Fig 1C; *F*_hormone type1,65_ = 187.23, *P* < 0.005; *F*_day7,65_ = 36.36, *P* < 0.005; *F*_interaction7,65_ = 16.40, *P* < 0.005). This pattern of change in serum levels of T_3_ and T_4_ was consistent with that observed in altricial birds in several previous studies [12–16].

When we analyzed the changes in thyroid hormone levels in males and females separately, total serum thyroid hormone levels (T_3_ plus T_4_) increased gradually in both sexes, with higher levels observed in males than in females during the course of post-hatching development from 10 to 30 dph (Fig 2A; *F*_group1,58_ = 92.48, *P* < 0.005; *F*_day6,58_ = 79.47, *P* < 0.005; *F*_interaction6,58_ = 2.35, *P* < 0.05). In the case of serum T_4_, the levels continually increased in both males and females from the time of hatching and accumulated until 40 dph. Serum T_4_ levels in males were higher than those in females during early post-hatching development from 10 to 20 dph (Fig 2B; *F*_group1,62_ = 20.51, *P* < 0.005; *F*_day6,62_ = 51.64, *P* < 0.005; *F*_interaction6,62_ = 2.20, ns). In the case of serum T_3_, male serum T_3_ levels were about two-fold higher than the levels recorded for females after 15 dph (Fig 2C; *F*_group1,61_ = 163.62, *P* < 0.005; *F*_day6,61_ = 30.84, *P* < 0.005; *F*_interaction6,61_ = 8.62, *P* < 0.005). No significant peaks were observed until 40 dph, which was clearly different from the patterns observed in precocial birds [15, 16].

There was clear augmentation from 25 to 35 dph in the total thyroid hormone levels (T_3_ plus T_4_) in the brain during development in both males and females, and the levels at the peak were almost the same in both sexes (Fig 3A; *F*_17,69_ = 75.88, *P* < 0.005). These results suggest that the uptake of total thyroid hormone from serum did not differ between the sexes. However, when we focused on the changes in brain T_3_ and T_4_ levels separately, the onset of the surge of T_4_ in males preceded that of females (Fig 3B), whereas the onset of the surge of T_3_ in males succeeded that of females (Fig 3C). At the peak, the T_3_ level in male brains was higher than that in females (Fig 3C). Brain T_4_ level in males started to increase at 15 dph, subsequently reaching a stable concentration at 20 dph that continued until 35 dph, and then decreased thereafter. Brain T_4_ in females started to increase at 20 dph, reached a stable level at about 5 days later than male T_4_, and then decreased in a similar way to that observed in males (Fig 3B; *F*_17,74_ = 75.97, *P* < 0.005). With regard to brain T_3_, it started accumulating in females just after hatching, whereas in males, its level remained low after hatching and did not start increasing until 15 dph, which was later than that observed in females. Female T_3_ then peaked around 20 to 25 dph and decreased at 40 dph, whereas male T_3_ peaked around 30 to 35 dph and decreased at 40 dph (Fig 3C; *F*_17,77_ = 75.64, *P* < 0.005). The data here show the differences in the onset of the surge of brain T_3_ levels between males and females. Considering the fact that the serum thyroid hormone levels increased constantly until 40 dph, the inflow rate of thyroid hormones from the blood into the brain may decline, or the turnover rate of the hormones in brain may accelerate between 35 and 40 dph.

**Fig 4:**
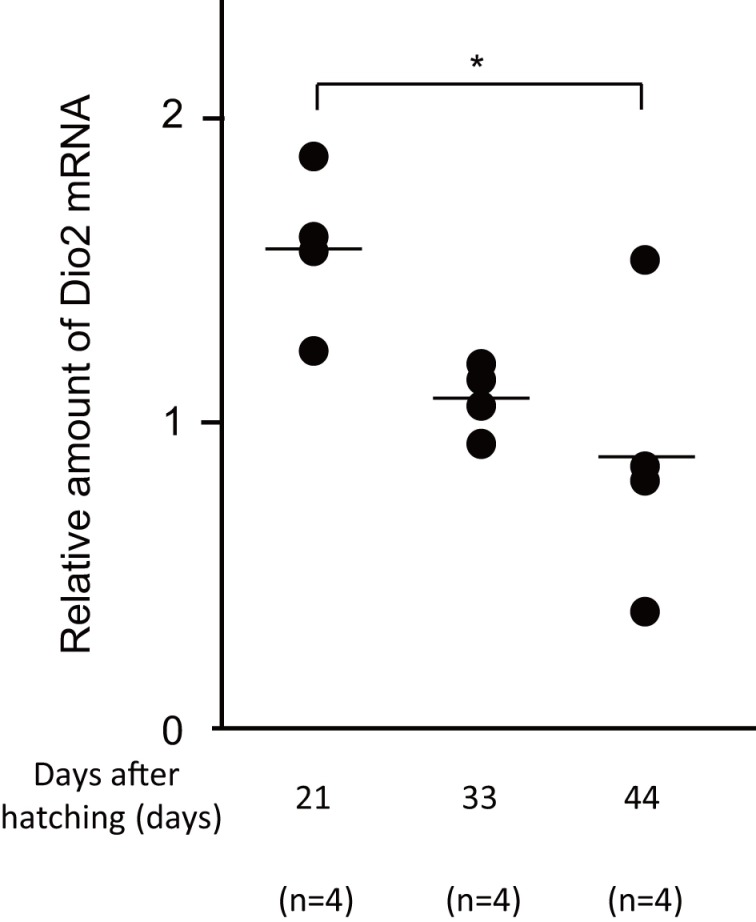
Dio2 mRNA levels in male zebra finches. Comparison of Dio2 mRNA levels in male zebra finches at 21 (n = 4), 33 (n = 4), and 44 dph (n = 4). The Kruskal-Wallis test and subsequent multiple comparisons were performed. The significance level was set at *P* < 0.05. Mean was shown in the graphs as a bar.

**Fig 5:**
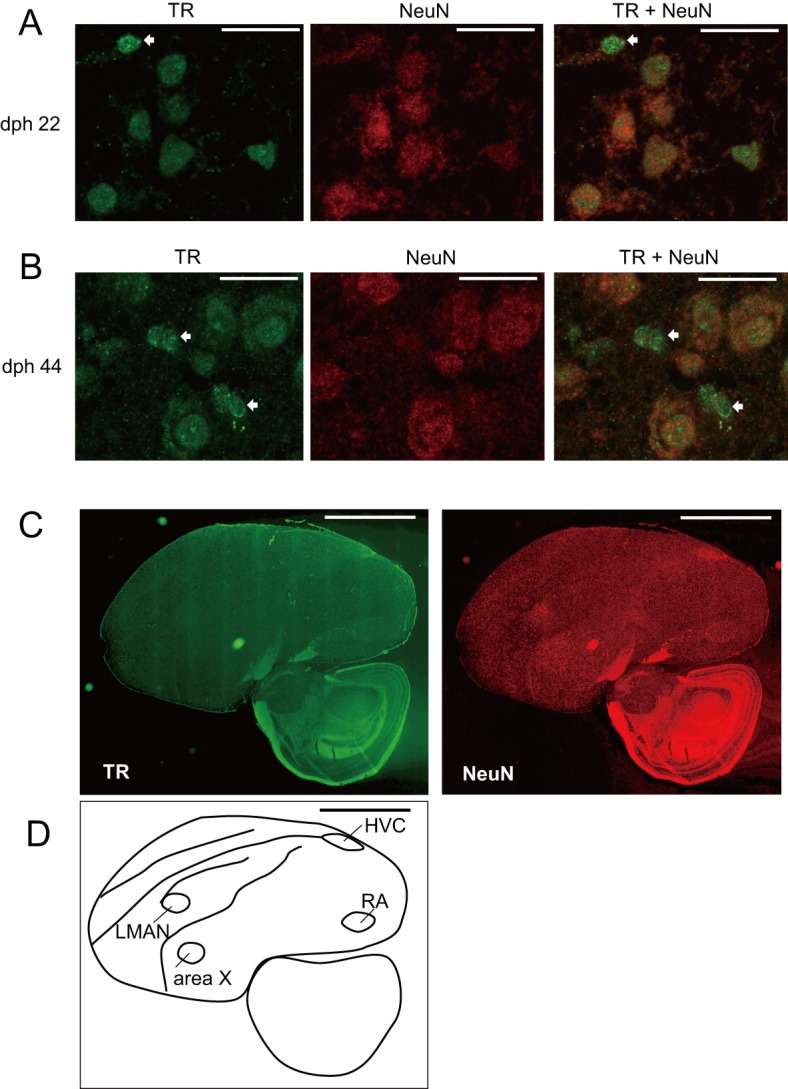
Thyroid hormone receptor in the male zebra finch brain. (A, B) Zebra finch male brain sections (A; dph 22, B; dph 44) were used for immunostaining. Labeling of thyroid hormone receptor (TR) alpha protein (left) and NeuN, a neuron specific marker protein (middle) in the same male zebra finch brain section. Images shown in the left and middle have been combined (right). Arrows show brain cells that were not labeled with NeuN. Bars in A and B indicate 25 μm. (C) Male zebra finch brain section (dph 44) was labeled by TR (left) and NeuN (right). (D) Schematic drawing of the section shown in C. Bars in C and D indicate 2.5 mm.

We next determined the Dio2 mRNA levels in male zebra finch brains to test whether Dio2 might be involved in the surges in T_3_ levels during development. We previously reported that Dio2 mRNA is upregulated in response to the imprinting training in chick brains [9]. In the presented study, using quantitative RT-PCR, we found the Dio2 mRNA level was higher at 21 dph and gradually decreased to 44 dph (Fig 4). This result suggested that Dio2 is involved in the surge of brain T_3_ during post hatch development. Just as the thyroid hormone receptor (TR) distributes in neuronal and glial cells in mammals [24], T_3_ in the zebra finch brain may act on the neuron and/or glia cells through its specific receptor. As expected, our immunofluorescence study showed that TR was distributed ubiquitously in the whole brain section and the signals were detected in both NeuN-positive and NeuN-negative cells (Fig 5A, 5B and 5C).

## Discussion

Here, we showed a clear difference in brain thyroid hormone levels during post-hatching development between precocial and altricial birds. In the precocial chick brain, thyroid hormone level peaked around hatching and then declined to the baseline level several days after hatching. In contrast, in altricial zebra finch, no clear perihatch peak was observed for the brain thyroid hormone and the changes in the levels such that they reached the maximum were delayed until 3–4 weeks post-hatching. We also found different dynamics in the levels of serum thyroid hormones between male and female zebra finches. Serum thyroid hormones were more abundant in males than in females during the early post-hatching development. This result suggests that thyroid hormones can be used for male-specific post-hatching development. T_4_ started accumulating in male brains earlier than it did in females; consequently, male brain T_4_ levels increased 5 days earlier than that observed in females (Fig 3B). Conversely, in the case of T_3_, the augmentation of female brain T_3_ preceded that of male brain T_3_ (Fig 3C). These results suggest that there is a difference in the onset of the surge between males and females. Considering the fact that the uptake of total thyroid hormone into the brain did not differ between the sexes (Fig 3A), the conversion of T_4_ to T_3_ by Dio2 was likely regulated in a sex-dependent manner, which would be an interesting topic to be explored in the future.

Our study aimed to address the question: What is the postnatal function of thyroid hormone in zebra finches? In addition to its role in cellular metabolism, thyroid hormone regulates neural development in mammals, such as neural cell proliferation, differentiation, migration, neurite growth, and synaptogenesis [1, 2, 25–27]. In avian species, expression studies about TR that evaluated deiodinase and TH-transporter during embryonic development have been previously conducted [28–30]. Because TR was ubiquitously expressed in the post-hatching zebra finch brain (Fig 5C), it is rational to assume that thyroid hormone has various functions in brain development. The total thyroid hormone level reached a stable value around 20−25 dph. Altricial birds typically show thermoregulatory responses and start feathering for thermal insulation around this period [15]. Thyroid hormones may help the development of zebra finches in these aspects and thus strengthen their locomotor activities, which may facilitate their fledging around 20 dph.

Furthermore, because brain T_3_ showed a difference in peak period between males and females, T_3_ may have a sex-specific function in zebra finches. We previously showed that in chick brains additional T_3_ is converted from T_4_, triggered by imprinting training; this is required for the execution of imprinting [9]. Rearing zebra finch chicks with their parents in nests inspired an intriguing hypothesis that some kind of learning behavior affected the sex-specific differences in T_3_ and T_4_ levels during post-hatching development. The learning behavior that affected the augmentation of T_3_ in female brains just after hatching remains to be ascertained; however, it is evident that female-specific differentiation in the brain may start at an early stage of the post-hatching development because some steroid hormones are known to have sex-specific effects on post-hatching development, even when chicks were exposed to the hormones at the embryonic stage [31]. As for male zebra finch chicks, they memorize the song of an adult “tutor” during 25–60 dph as a critical period of “sensory” learning [17]. Dio2 mRNA level was higher at 21 dph and gradually decreased to 44 dph (Fig 4). In addition, a recent study found that Dio2 mRNA is abundant in the sensory learning phase in male song control nuclei such as Area X, HVC and the robust nucleus of the arcopallium (RA) in zebra finch [32], suggesting a role for thyroid hormone in the development of the song control nuclei during the course of song learning. The “sensorimotor” learning period starts around 30 dph, when they start singing and gradually match their initially immature vocalizations to the memorized song. Therefore, in the zebra finch, 'sensory' and 'sensorimotor' learning phases are reciprocally affected during their development at around 30–50 dph. After this learning process, the song in adults usually remains unchanged or 'crystallized' [33]. Thyroid hormone in a zebra finch male brain may be involved in the sensory and/or the sensorimotor learning around the merged learning phase. Our data show the correlation between thyroid hormone level and sex differences in the development of song system. Establishing those connections would be a topic to be addressed in the future.

In conclusion, our study shows for the first time that brain thyroid hormone levels in altricial birds increase markedly around 20–35 dph, which is in contrast to the patterns observed in precocial birds. The differences in these patterns may reflect some aspects of differences in the learning behavior between precocious and altricial species.

## Supporting Information

S1 Table List of raw data for Figs 1–3. (XLSX)

S2 Table List of raw data for Fig 4. (XLSX)
